# Analysis of the Genetic Diversity and Genetic Structure of Bamei Pig Population Based on Single-Nucleotide Polymorphism Chip

**DOI:** 10.3390/ani16142227

**Published:** 2026-07-18

**Authors:** Pengfei Wang, Qiaoli Yang, Zunqiang Yan, Xi Long

**Affiliations:** 1 College of Animal Science and Technology, Gansu Agricultural University, Lanzhou 730070, China; wangpf@gsau.edu.cn (P.W.); yangql@gsau.edu.cn (Q.Y.); 2 Chongqing Academy of Animal Sciences, Chongqing 402460, China

**Keywords:** Bamei pig, ROH, inbreeding coefficient, preservation of species

## Abstract

This paper assessed the genetic diversity, structure, and distance of Bamei pigs, a traditional Chinese indigenous breed, to conserve their genetic resources and facilitate comprehensive production planning. Using a 50K single-nucleotide polymorphism (SNP) chip, we genotyped 64 individuals from a Bamei pig conservation farm. Based on these data, we calculated genetic diversity parameters, constructed family structures, and identified runs of homozygosity (ROH) for each individual to obtain corresponding inbreeding coefficients. The results showed that the Bamei pig conservation population had an average genetic distance of 0.2707. The population maintained relatively rich genetic diversity, as indicated by a polymorphic marker proportion of 88.28% and observed (0.351) and expected (0.340) heterozygosity values that were very close. The inbreeding coefficient calculated based on ROH was at a medium level. The average inbreeding coefficient was 0.1302. Substantial inter-individual variation was observed, with some individuals showing relatively high inbreeding coefficients. Kinship analyses further confirmed the presence of close genetic relationships among certain individuals within the population and potential inbreeding risks. Family-structure analysis classified the boars into six families, with an uneven distribution of boars and sows both across and within these families. In order to ensure the continuous protection of the breed, it is urgent to optimize the breeding program. In the process of offspring retention, individuals with distant genetic relationships with boars and sows should be selected as much as possible, the inbreeding coefficient of the population should be gradually reduced, the number of families should be paid attention to, and the proportion and structural balance of each family should be paid attention to.

## Simple Summary

This study assessed the genetic diversity, structure, and inbreeding of Bamei pigs using a 50K SNP chip on 64 individuals. The population showed relatively rich genetic diversity and low average inbreeding, but with high individual variation. Family-structure analysis revealed six families with uneven sex distribution, posing a lineage-loss risk. While current conservation efforts have largely maintained pure germplasm and diversity, refined mating schemes and possible exogenous-gene introduction are needed to mitigate persistent inbreeding risks.

## 1. Introduction

China has abundant pig resources in the world, and local pig breeds account for 30% of the world’s pig resources [1]. However, the protection and development of local pig breed resources are faced with the huge challenges of population reduction and the introduction of pig-breed gene infiltration [2]. The Bamei pig is an ancient indigenous breed of the meat-and-lard type, primarily distributed in Northwest China (such as Gansu, Ningxia, Qinghai), and it belongs to the North China type [3]. Characterized by strong adaptability, roughage tolerance, and fresh-tasting meat, the Bamei pig serves as a valuable maternal resource for producing specialty pork and conducting hybrid utilization [4,5]. The relatively long growth cycle of the Bamei pig has made it difficult to meet the demands of breeding profitability. As a result, under the influence of a market economy, farmers have mostly shifted to introducing commercial breeds, leading to a continuous decline in Bamei pig populations, loss of genetic diversity, and erosion of valuable genetic resources. It is necessary to formulate a scientific protection plan for Bamei pigs. In response, Bamei pig conservation farms were established to gradually expand the conservation population, and genetic diversity was enriched through the introduction of breeding stock from conservation farms in Qinghai, Shaanxi, and other regions. Previous studies have revealed the genetic diversity of Bamei pigs and the infiltration degree of commercial pig breeds by using a sequence of mitochondrial DNA D-loop region. However, mitochondrial markers only reflect maternal genetic information, and it is difficult to comprehensively evaluate the genetic variation and population structure at the genome level [6]. However, the current status of genetic variation and the population composition of the conserved population remain unclear, hindering the advancement of scientifically informed conservation efforts. Therefore, it is essential to assess the conservation effectiveness on a molecular level.

The declining cost of genome-wide genotyping in recent years has made it an increasingly accessible and valuable tool for investigating the genetic characteristics of livestock (including cattle, pigs, goats, sheep and chickens) and accurately analyzing the genetic structure, genetic relationship and inbreeding level of the population on the genomic level [7,8]. Single-nucleotide polymorphisms (SNP) chips have been frequently used to examine genetic diversity in several indigenous pig breeds (such as Liangshan pigs [9], Min pigs [10], Tongcheng pigs [11] and Licha black pigs [12]) in China. The effect of conservation was evaluated, which provided theoretical support for the scientific formulation of conservation strategies. For example, Liu et al. [9] analyzed the genetic structure of 139 Liangshan pigs by SNP chip, and found that the mean genetic distance was 0.2823. A total of 983 runs of homozygosity (ROH) were calculated, 80% of which were shorter than 100 Mb. The population can be partitioned into five families, with an effective population size (Ne) of 15, suggesting that Liangshan pigs exhibit limited genetic diversity.

In this paper, a 50K SNP chip was utilized to assess the genetic diversity and relationships, population composition, and inbreeding coefficients of a Bamei pig population maintained in a conservation farm. The findings from this study have the potential to make scientific management and conservation strategies for this breed.

## 2. Materials and Methods

### 2.1. Animals and DNA Extraction

According to the existing pedigree records of six boar families in Lingtai County Wang Fu Xin Bamei Pig Breeding Pig Farm from Gansu province, 5–6 adult boars and 4–5 adult sows were randomly selected from each family. A total of 64 (34 boars, 30 sows) ear tissue samples were collected for DNA extraction. Genomic DNA was isolated using the CWE9600 Magbead Blood DNA Kit (Kangwei Century Biotechnology Co., Ltd., Taizhou, China). The extracted DNA integrity and purity were detected using a Nanodrop 2000 nucleic acid protein analyzer (Thermo Scientific, Waltham, MA, USA) and 0.8% agarose gel electrophoresis (170 V, 25 min). In this study, the ratio of OD260 to OD280 of DNA was between 1.7 and 2.1, with an individual sample concentration exceeding 50 ng/µL. Subsequently, the DNA samples were preserved at −20 °C pending further analyses.

### 2.2. SNP Detection and Data Quality Assessment

Genomic DNA samples that passed quality inspection were genotyped using the “Zhongxin-1” 50K SNP chip developed by Beijing Compass Biotechnology Co., Ltd. (Compass), Beijing, China. Qualified gDNA (concentration ≥ 50 ng/uL) was subjected to genome-wide amplification of all samples and incubated at 37 °C for 24 h, followed by gDNA fragmentation, precipitation, and re-suspension in hybridization buffer. The re-suspended DNA fragments were applied onto the chip and incubated at 48 °C for 16 h to facilitate hybridization. Following this step, a washing procedure eliminated non-specifically bound DNA, and the remaining sites exhibiting specific binding subsequently underwent single-base extension. Then, the iScan Reader was used to scan and obtain original data, and quality control was performed using PLINK (v1.90). Based on the criteria established in previous studies [10,11], we filtered out unqualified SNPs and samples according to the following standards from Compass: first, only autosomal SNPs were retained; second, SNP call rate ≥ 90%; third, individual call rate ≥ 90%; fourth, SNPs with minor allele frequency (MAF) < 0.01 were removed; and fifth, Hardy–Weinberg equilibrium *p*-value ≥ 0.000001. Additionally, to ensure analytical accuracy and prevent the influence of repeated samples on the gene frequency, PLINK (v1.90) was employed to identify duplicate samples before the analysis. The criterion for the determination of repeated samples is DST ≥ 0.99 between samples, in which case it is judged as a repeated sample.

### 2.3. Genetic Diversity Analysis

The concept of Ne is taken to be the size of an ideal population that would exhibit the same rate of change in gene-frequency variance or inbreeding coefficient as the population under study [13]. The Ne was derived from the following equation: Ne = (1/4c) × (1/r^2^ − 1). In this work, Ne was estimated using a linkage disequilibrium-based approach implemented in the SNeP (v1.1) [13]. The frequency of polymorphic loci in the target population, termed the polymorphic marker ratio (P_N_), was also obtained. PLINK (v1.90) [14] was used to calculate the minimum allele frequency for each locus, followed by a custom R script to compute the final P_N_ value. The PN was estimated using the formula PN = M/N, with M representing polymorphic loci and N representing the total loci. Furthermore, two standard measures of genetic diversity were assessed: expected heterozygosity (He), which denotes the probability that a randomly selected individual is heterozygous at a given locus, and observed heterozygosity (Ho), representing the actual heterozygous proportion at a locus. These values, calculated with PLINK (v1.90), provide insights into population. Specifically, Ho < He is suggestive of inbreeding or recent selection, whereas Ho > He may indicate the introduction of genetic material from an external population.

### 2.4. Inbreeding Coefficient Calculation

A certain number and density of SNPs on the genome appear to be homozygous regions, that is, ROH, which is an unbroken region of homozygosity in individuals [15]. PLINK (v1.90) was employed to detect the ROH of the Bamei pig population, and the number, length and distribution of ROH in each sample were counted. The criteria for identifying ROH were as follows from Compass: first, the minimum length of ROH is 1000 kb; second, the maximum distance between adjacent SNPs in ROH is less than 1000 kb; third, the minimum density of SNPs in ROH is 100 kb per SNP; fourth, the sliding window consists of 50 SNPs, and each slide moves by 1 SNP; fifth, the missing rate in a single sliding window is ≤1 and the number of heterozygotes is ≤1; sixth, the window threshold is 0.05; and seventh, each ROH is composed of at least 30 SNPs. The total length of the pig autosomal genome is approximately 2,449,080.462 kb. The ratio of the total ROH fragment length relative to the entire autosomal genome length in each individual was calculated. That is the inbreeding coefficient based on ROH.

### 2.5. Genetic Relationships and Population-Structure Analysis

Genetic kinship among individuals was assessed using GCTA (v1.94) [16], with the resulting relatedness estimates subsequently visualized through heat maps. Then, an identity-by-state (IBS) distance matrix was generated utilizing PLINK (v1.90). This matrix served as the foundation for clustering analysis to elucidate population structure, which was performed via the neighbor-joining (NJ) method and graphically represented using Mega X (v10.0) [17]. Combined with the results of genomic genetic-relationship analysis, the genomic genetic-relationship coefficient between boars ≥ 0.1 was used as the basis for clustering, and then the genetic relationship between sows and boars was used to classify and construct the Bamei pig conservation population.

## 3. Results

### 3.1. SNP Typing and Quality-Control Analysis

**Figure 1:**
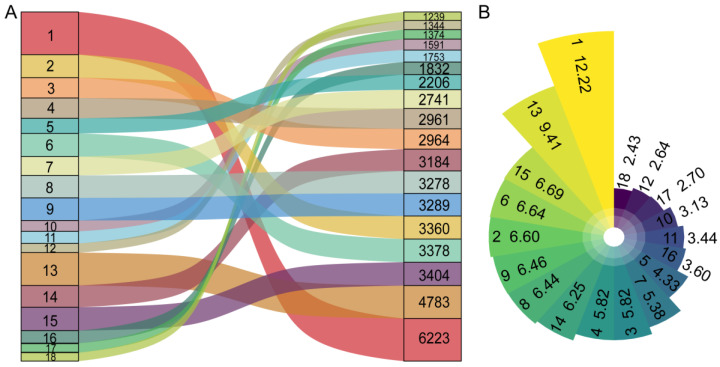
Distribution diagram (**A**) and scale diagram (**B**) of SNP before quality control. The left side represents the chromosome number, and the right side represents the number of SNP. The outer side represents the chromosome number, and the inner side represents the proportion of SNP number.

**Figure 2:**
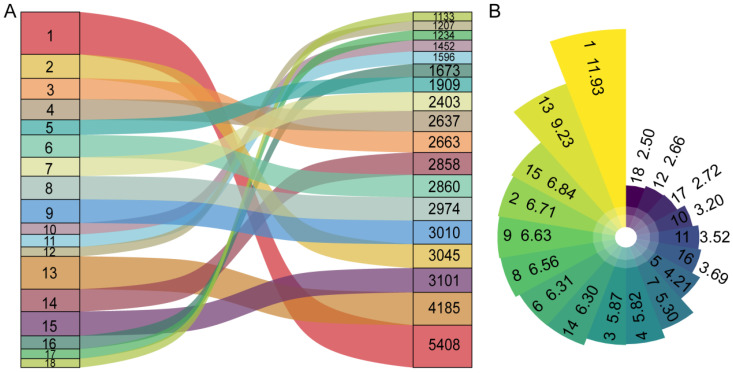
Distribution diagram (**A**) and scale diagram (**B**) of SNP after quality control. The left side represents the chromosome number, and the right side represents the number of SNP. The outer side represents the chromosome number, and the inner side represents the proportion of SNP number.

**Figure 3:**
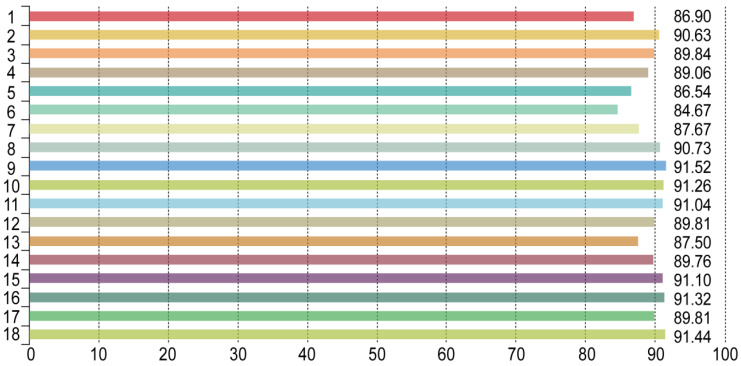
The bar chart of the pass rate of SNP quality control on chromosomes.

The genomic DNA samples of Bamei pigs were genotyped by “Zhongxin-1” chip, and the quality control of data was carried out. After quality control, duplicate samples were not identified in this paper. All samples exhibited detection rates of at least 90%. A total of 34 boars and 30 sows were left for subsequent analysis. It was found that there were 4649 markers with MAF < 0.01, 858 markers with SNP detection rate < 0.90, 43 markers with Hardy–Weinberg equilibrium test *p* < 10^−6^, six insertion/deletion markers, and 45348 markers after quality control (Table 1). The “Zhongxin-1” array contains 57466 markers, of which 50904 SNPs were successfully detected in the Bamei population before quality filtering. The number of SNPs on chromosome 1 was the highest, 6223, accounting for 12.22%, and the number of SNPs on chromosome 18 was the least, 1239, accounting for 2.43% (Figure 1). After quality control, there were a total of 45348 SNPs. The number of SNPs on chromosome 1 was the largest, which was 5408, accounting for 11.93%, and the number of SNPs on chromosome 18 was the least, which was 1133, accounting for 2.50% (Figure 2). The total SNP after quality control accounted for 89.09% of the total SNP before quality control. The pass rate of SNP quality control on chromosomes was higher than 84%. The pass rate of SNP quality control was highest on chromosome 9 at 91.52% and lowest on chromosome 6 at 84.67%, respectively (Figure 3). The marker density after quality control is 18.52 SNPs/Mb.

### 3.2. Genetic Diversity Analysis

**Figure 4:**
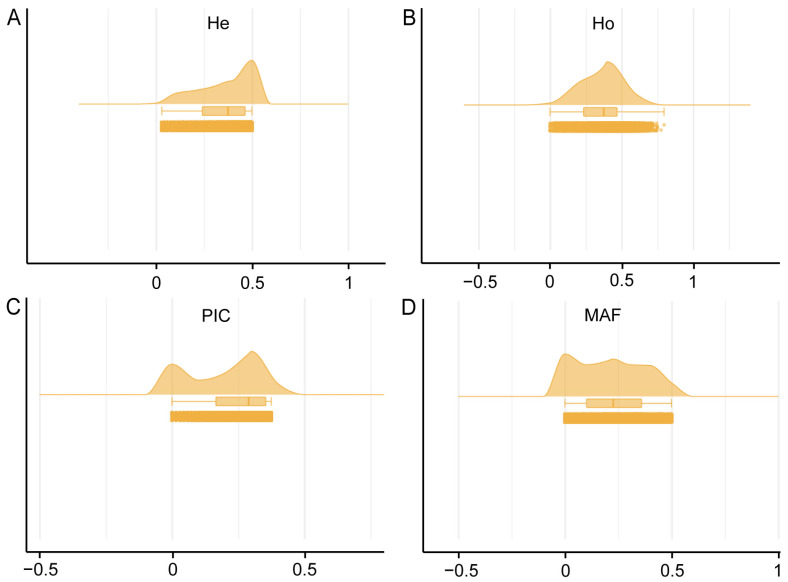
Analysis of the genetic diversity of the Bamei pig population. (**A**) Raincloud plot for He. (**B**) Raincloud plot for Ho. (**C**) Raincloud plot for PIC. (**D**) Raincloud plot for MAF.

**Figure 5:**
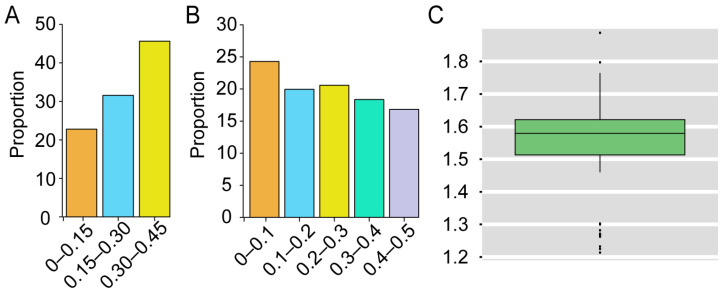
Analysis of PIC, MAF ratio and effective allele number in the Bamei pig population. (**A**) Polymorphic information content ratio bar chart. (**B**) MAF ratio bar chart. (**C**) Box plot of effective allele number.

The mean value of Ne and P_N_ of the Bamei pig population were 3.2 and 0.8390, respectively (Table 2). The average value of He was 0.3400, with a maximum of 0.5000 and a minimum of 0.0308 (Table 2, Figure 4A). The average value of Ho was 0.3512, the maximum value was 0.7969, and the minimum value was 0 (Table 2, Figure 4B). The average value of polymorphism information content (PIC) was 0.2476, of which 0~0.15 accounted for 22.80%, 0.15~0.30 accounted for 31.57%, 0.30~0.45 accounted for 45.63%, and the maximum value was 0.3750, while the minimum value was 0 (Table 2, Figure 4C and Figure 5A). The average MAF was 0.2293, 0~0.1 accounted for 24.30%, 0.1~0.2 accounted for 19.95%, 0.2~0.3 accounted for 20.57%, 0.3~0.4 accounted for 18.36%, 0.4~0.5 accounted for 16.82%, and the maximum value was 0.5000, while the minimum value was 0 (Table 2, Figure 4D and Figure 5B). The average effective number of alleles was 1.5576, the maximum value was 1.8879, which belonged to individual 6-4, and the minimum value was 1.2136, which belonged to individual 2-1 (Table 2, Figure 5C).

### 3.3. Molecular Kinship Analysis

**Figure 6:**
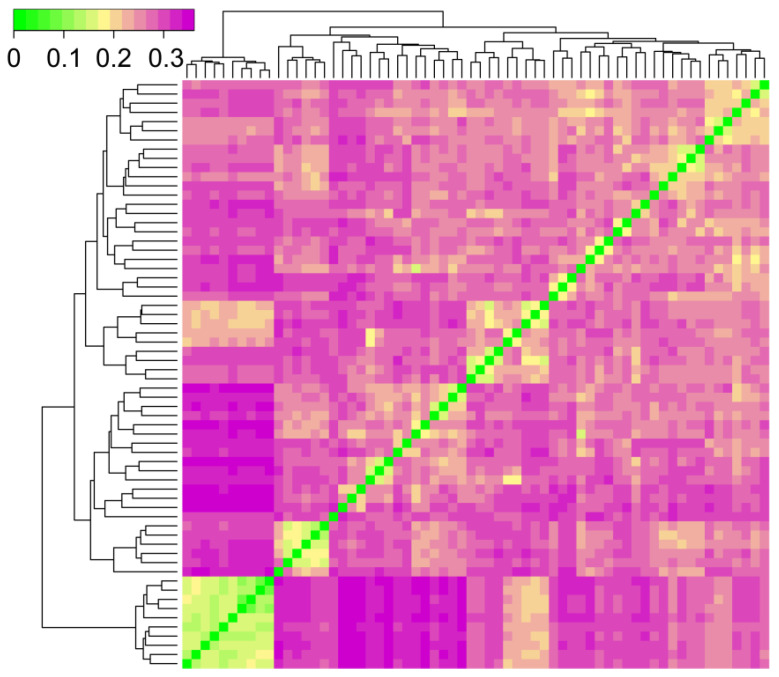
The visual representation of the IBS distance matrix results. In the IBS distance matrix, each cell corresponds to the genetic distance between a pair of samples, arranged sequentially from the first to the last. Larger values are represented by a redder color, indicating a greater genetic distance between the two individuals, whereas smaller values indicate a closer genetic relationship.

**Figure 7:**
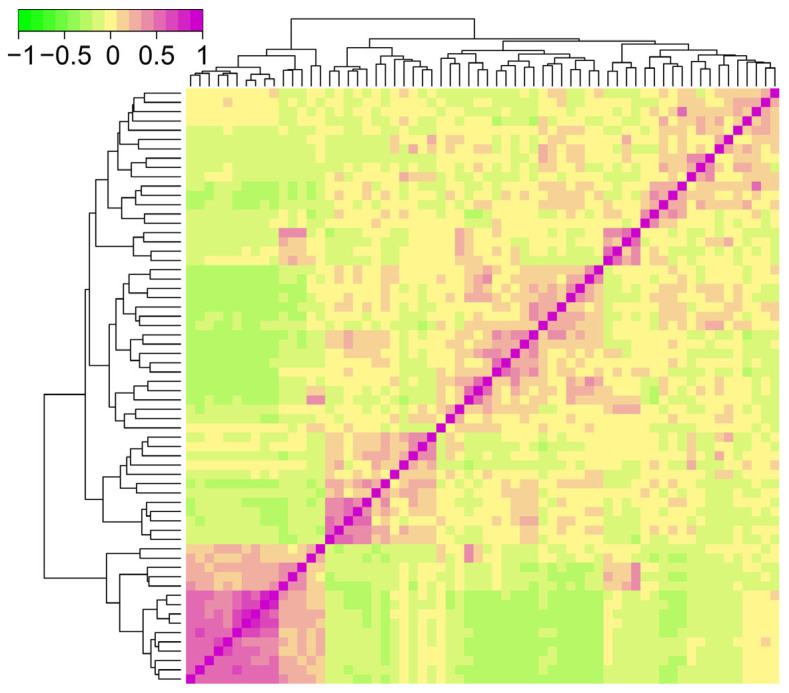
The visual representation derived from the G matrix. In the resulting G matrix, each small square denotes the relationship coefficient between a pair of individuals, ordered sequentially from the first to the last sample. Higher values correspond to a redder color, indicating a closer genetic relationship between the two individuals.

The results of IBS genetic distance were displayed in Figure 6. The IBS genetic distance of individuals in the Bamei pig population was 0.0723~0.3609, and the mean genetic distance was 0.2707, indicating that there was a far genetic distance between Bamei pig individuals. IBS matrix analysis showed that most individuals had a far genetic distance and a far genetic relationship, and a small number of individuals exhibited a close genetic distance and kinship, indicating that there was an inbreeding risk within the Bamei pig population. The results of G-matrix analysis were shown in Figure 7. The genetic relationship of individuals in the conservation population of Bamei pigs was −0.3302~0.7975, and the average genetic relationship was −0.0158. The genetic relationship between most individuals was far, and the genetic relationship between a few individuals was close, which corroborated the findings of the IBS distance matrix analysis.

### 3.4. Inbreeding-Coefficient Analysis Based on ROH

**Figure 8:**
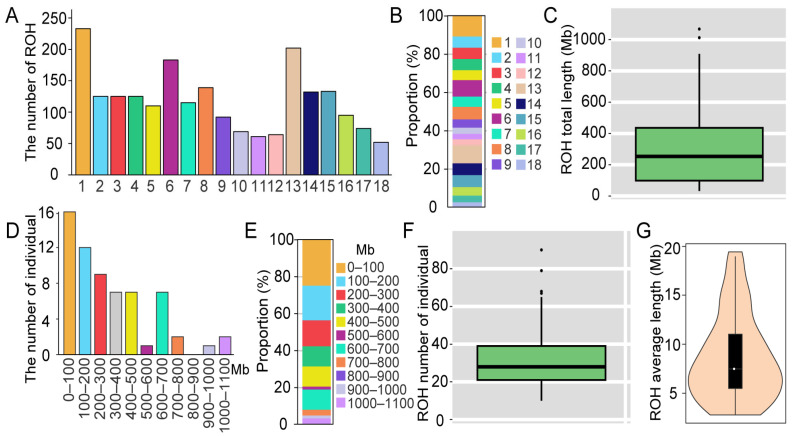
The number and proportion of ROH in Bamei pigs. (**A**) The number of ROH on different chromosomes. (**B**) Stacking map for the distribution ratio of ROH on different chromosomes. (**C**) Box plot for ROH total length distribution of individuals. (**D**) Individual number bar graph corresponding to different total lengths of ROH. (**E**) Individual proportion stacking diagram for different total lengths of ROH. (**F**) Box plot for ROH number of individuals. (**G**) Violin plot for ROH average length of individuals.

The distribution number and proportion of ROH on 18 autosomes were counted. The results showed that there were 2129 ROH. The number of ROH on chromosome 1 was the largest (233), accounting for 10.94%. The number of ROH on chromosome 13 was 202, accounting for 9.49%. The number of ROH on chromosome 11 was 61, accounting for 2.87%, while the number of ROH on chromosome 18 was the least (52), accounting for 2.44% (Figure 8A,B). The distribution of the ROH total length of individuals was analyzed. The ROH total length of the Bamei pigs with the maximum value was 1067.41 Mb (2-1 individual) and the minimum value was 32.1551 Mb (6-4 individual). The number of individuals with ROH total length within 0~100 Mb was 16, accounting for 25%. The number of individuals with ROH total length within 300~400 Mb, 400~500 and 600~700 Mb was seven, each accounting for 10.94%. Very few individuals had ROH lengths falling within the 500–600 Mb and 900–1000 Mb ranges, only 1, each accounting for 1.56% (Figure 8C–E). The number of ROH of each individual was analyzed. The largest number of ROH belonged to the 1-6 individual, which was 90, and the smallest number of ROH belonged to the 2-10, 6-4, 5-6 and 3-7 individuals, all of which had ROH number 10 (Figure 8F). The average ROH length of each individual ranged from 2.79 to 19.41 Mb. Individuals 2-1 and 6-8 had the maximum average value and the minimum average value, respectively (Figure 8G).

**Figure 9:**
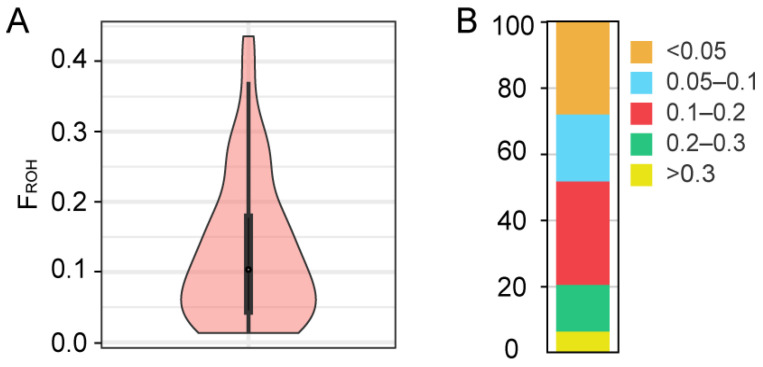
Violin plot (**A**) and stacking diagram (**B**) of the inbreeding coefficient F_ROH_.

By counting the ROH length of all individuals in the Bamei pig population, the inbreeding-coefficient value according to the individual ROH length was obtained. Figure 9A,B showed the visualization results of the inbreeding coefficient based on the ROH length, and showed the inbreeding-coefficient distribution of all samples. The average inbreeding coefficient of the population (64 individuals) is 0.1302, and the individual with the largest inbreeding coefficient is 2-1, which is 0.4358. The individual with the smallest inbreeding coefficient is 6-4, which is 0.0131. In addition, the average inbreeding coefficients calculated by ROH lengths were F_ROH_ > 10 Mb = 0.2359 (20 individuals), F_ROH_ 5–10 Mb = 0.10884 (30 individuals), and F_ROH_ 0–5 Mb = 0.0249 (14 individuals). There are 18 individuals with inbreeding coefficient less than 0.05, accounting for 28.13%. There are 13 individuals with inbreeding coefficient between 0.05 and 0.1, accounting for 20.31%. There are 20 individuals with inbreeding coefficient between 0.1 and 0.2, accounting for 31.25%. There are nine individuals with inbreeding coefficient between 0.2 and 0.3, accounting for 14.06%. There are four individuals with inbreeding coefficient greater than 0.3, accounting for 6.25%.

### 3.5. Construction of Family Groups

**Figure 10:**
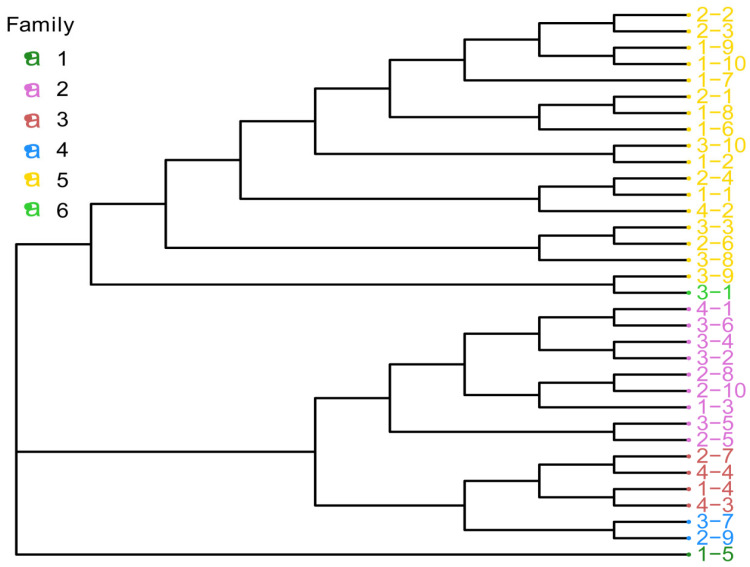
Clustering analysis of boars in the Bamei pig conserved population.

Considering the critical role that boars play within the entire conservation population, this study first used the neighbor-joining method to cluster the boar population based on the standard of the molecular genetic relationship coefficient among boars being no less than 0.1. The results are shown in Figure 10. Finally, 34 boars were divided into six families, with one boar in Families 1 and 6, and 17 boars in Family 5. According to the degree of genetic relationship between sow individuals and different family boars, the sows were classified into different families (Table 3). The Bamei pig conservation population was within the family of the boar, and there was no other family that did not have a blood relationship with the boar. The Bamei sows in some families had crossover phenomenon at the same time and were divided into six different families. For example, sow 5-2 was divided into Family 1, Family 3 and Family 4 at the same time, and sow 6-9 was divided into Family 1, Family 2, Family 4 and Family 5 at the same time.

## 4. Discussion

### 4.1. The Seed Industry and Bamei Pigs

Bamei pigs, as an essential component of China’s livestock genetic resources, are mainly distributed across Pingliang City in Gansu Province, Yulin City in Shaanxi Province, and Huzhu County in Qinghai Province [18]. Nevertheless, the population of Bamei pig has been experiencing a persistent decline in recent years. To facilitate effective conservation and sustainable development of this breed, the present study performed a thorough assessment of population structure, genetic diversity, ROH, linkage disequilibrium, and Ne via commercial SNP chip. Elucidating the population structure and genetic diversity of Bamei pigs is fundamental to formulating management strategies and preserving their excellent traits.

### 4.2. Genetic Diversity

Assessing the Ne of the current population through genotype data has emerged as a critical research subject within the field of conservation genetics and a reduction in the Ne leads to decreased genetic diversity, a condition that is unfavorable for subsequent genetic advancement [19,20]. The Ne of the Bamei pig population was 3.2, which was lower than that of the Licha black pig (8.7) [12], the Jiangshan black pig (4.9) [21], the Liangshan pig (15) [9], the Luopanshan pig (4.1) [22], the Penzhou mountain pig (3.5) [22], the Rongchang pig (4.6) [22], the Jinhua pig (96) [23] and the Fengjing pig (6.8) [24], but higher than that of the Hechuan black pig (2.9) [22] and the Quxi pig (2.7) [22]. This is likely due to the limited population size, high inbreeding level, and closed nucleus breeding scheme, which have reduced genetic diversity in population. The P_N_ of Bamei pig population was 0.839, which was higher than that of the Licha black pig (0.827) [12], the Min pig (0.663) [10] and the Fengjing pig (0.469) [24], but lower than that of Chinese indigenous pigs (such as the Jiaxinghei pig) in Zhejiang Province [25]. This variation may stem from differences in sample sizes and the specific computational methodologies employed. Heterozygosity is a key indicator to measure genetic variation and the higher the heterozygosity of the population, the richer the genetic diversity [26]. In this study, Ho (0.351) was slightly higher than He (0.340), indicating that a very small part of the Bamei pig population may have crossbred with other pig breeds. Therefore, it is necessary to eliminate non-purebred breeds and purify the bloodlines. The He of the Bamei pig was lower than that of the Licha black pig (He = 0.3576) [12], the Xiangyang black pig (He = 0.360) [27], and higher than that of the Fengjing pig (He = 0.287) [24], the Min pig (He = 0.330) [10], and the Tongcheng pig (He = 0.310) [11]. This also indicates that an increasing number of SNP loci are tending towards homozygosity, diversity is decreasing, and there may be a risk of inbreeding. PIC is a quantitative index primarily used to evaluate the effectiveness of genetic markers in linkage analysis and to assess the level of genetic diversity in a population [28]. The higher the PIC value, the higher the gene locus. The average PIC of the Bamei pig population was 0.248. The PIC of most loci in the genome of the Bamei pig was between 0.30 and 0.45, accounting for 45.63%. The results showed that the Bamei pig has certain genetic diversity.

### 4.3. Genetic Distance and Population Structure

To assess genetic background diversity within the Bamei pig population, we constructed a NJ tree relying on the IBS distance matrix. The tree classified 34 pigs into six distinct families, revealing an unbalanced distribution of boars across families. Notably, two families contained only two boars each, highlighting potential risks of unequal family representation and inbreeding. Similar to our results, Liangshan boars were divided into five families using genotype data, with only two boars in three families [9]. Licha boars were also divided into eight different families via phylogenetic tree, with only two boars in one family [12]. Jiangshan black pigs were divided into four different families, and with four boars in one family [21]. It can be seen that there are not enough pigs to form six families of boars in some local pig breeds. There are six families of Bamei pigs in this study, which meet the requirements of at least six families in the local breed conservation farm. Additionally, the crossover phenomenon of sows in some families has been confirmed by many studies [21,29]. Similar to these papers, some sows existed in multiple families in this study. Our findings underscore the need for careful mating strategies to preserve bloodlines and maintain a balanced family structure in Bamei pig future conservation efforts. Using SNP chip data to elucidate effective relationships among Bamei pig breeding boars can inform selective breeding and guide the creation of frozen semen banks.

### 4.4. Inbreeding Coefficient

Inbreeding is the main cause of the reduction in genetic diversity in conservation populations. In traditional conventional breeding, incomplete or missing pedigree records can lead to a decrease in the accuracy of inbreeding coefficients, thereby affecting the conservation effect. The inbreeding coefficient derived from F_ROH_ exhibits greater accuracy than that obtained from pedigree data [30]. Thus, F_ROH_ in the Bamei pig population was measured. There were 20 individuals with inbreeding coefficients between 0.1 and 0.2, accounting for 31.25%. Among all Bamei individuals, the average inbreeding coefficient was 0.1302, which was lower than that of the Beijing black pig (0.1906) [31], the Wannan black pig (0.5234) [32] and the Fengjing pig (0.3180) [24], but was higher than that of the Liangshan pig (0.026) [9], the Licha black pig (0.110) [12] and the Jiangshan black pig (0.2530) [21]. This finding suggested that, despite the relatively limited core population size of the Bamei pig, the current breeding strategies have been able to mitigate inbreeding to some degree. The length of a homozygous fragment is determined by the generational distance to a common ancestor, with shorter fragments indicating a more distant ancestral origin [15,33]. In this paper, the ROH fragment lengths of the Bamei pig were predominantly found to be 0 Mb~100 Mb. It was hypothesized that this breed exhibits a low incidence of inbreeding behavior. A greater total length of ROH in an individual corresponds to a higher inbreeding coefficient [15,34]. Indeed, the maximum total length of ROH was 1067.41 Mb (individual 2-1), and the minimum total length of ROH was 32.16 Mb (individual 6-4). They have the largest and smallest inbreeding coefficients (0.4358 and 0.0131), respectively. In future production, for individuals with high F_ROH_ values, efforts should be made during offspring breeding to select boars that have a relatively distant genetic relationship with the sows, thereby gradually reducing the inbreeding coefficient.

This study has the following two limitations: first, the samples collected are only from the Bamei pig breeding farm in Gansu Province, and samples from the Bamei pig breeding farm in Shaanxi Province and Qinghai Province were not collected; second, the collected samples did not cover all populations of Bamei pigs. Subsequently, we plan to expand the sampling population for further research.

## 5. Conclusions

Based on the 50K SNP chip data, this study systematically evaluated the conservation effects of Bamei pigs from the perspective of population genetics. This study reveals that the conserved Bamei pig population maintains moderate genetic diversity but faces a relatively high inbreeding risk. Compared with other indigenous breeds, its effective population size (Ne = 3.2) and PIC (0.248) are lower, while the inbreeding coefficient (F = 0.1302) is higher, indicating an elevated risk of inbreeding depression and potential population degradation. The number of boars per family is limited (Family 6 has only one), necessitating enhanced breeding and selective retention from these families. Preference should be given to mating individuals with distant genetic relationships to gradually reduce the inbreeding coefficient. Additionally, balanced seed retention across families, introduction of new bloodlines, preservation of gametes and somatic cells, and regular genetic monitoring are recommended to safeguard genetic diversity.

## Figures and Tables

**Table 1:** Result of SNP quality-control statistics.

Quality-Control Standard	Numbers of SNPs
Total number of SNPs	57466
SNP with MAF < 0.01	4649
SNP with Hardy–Weinberg equilibrium *p* < 10^−6^	43
SNP with callrate < 0.90	858
SNPs on chromosome X	4252
SNPs on chromosome Y	2310
Insertion/deletion	6
SNPs used after quality control	45348

**Table 2:** Genetic diversity numerical value of the Bamei pig conserved population.

Type	Mean Value
Effective population size (Ne)	3.2
Polymorphic marker ratio (P_N_)	0.8390
Expected heterozygosity (He)	0.3400
Observed heterozygosity (Ho)	0.3512
Polymorphic information content (PIC)	0.2476
Minor allele frequency (MAF)	0.2293
Numbers of effective alleles	1.5576

**Table 3:** Family construction results of the sow conservation population of Bamei pigs.

Family Name	Number	ID
Family 1	19	4-10, 5-8, 5-7, 7-1, 5-2, 4-9, 7-4, 5-4, 5-3, 6-2, 6-3, 6-1, 6-9, 4-6, 5-10, 6-8, 6-10, 5-5, 4-8
Family 2	26	4-10, 5-8, 4-6, 4-8, 6-10, 6-7, 6-8, 5-5, 5-7, 7-1, 5-2, 4-9, 5-3, 5-9, 5-4, 7-4, 4-5, 5-1, 6-4, 6-2, 5-10, 6-3, 6-1, 4-7, 7-2, 6-9
Family 3	21	4-10, 5-8, 5-7, 7-1, 5-2, 4-9, 5-1, 6-4, 5-10, 6-8, 6-7, 5-9, 6-10, 5-4, 4-8, 5-3, 5-5, 7-4, 4-5, 6-2, 4-6
Family 4	21	4-10, 5-8, 5-7, 7-1, 5-2, 4-9, 6-2, 5-10, 6-8, 6-3, 6-1, 4-8, 4-7, 7-2, 6-9, 5-6, 5-1, 4-6, 6-10, 7-4, 5-5
Family 5	22	4-10, 5-8, 6-5, 6-6, 7-3, 7-2, 6-2, 5-10, 6-8, 6-3, 6-1, 4-8, 4-7, 6-9, 6-4, 4-6, 7-4, 5-6, 5-1, 6-10, 5-5, 6-7
Family 6	3	5-9, 4-9, 6-10

Note: The samples that appear in multiple families at the same time are cross-family samples, that is, the individual is closely related to some samples in these families.

## Data Availability

The original contributions presented in this study are included in the article. Further inquiries can be directed to the corresponding authors.
